# Hypoxia promotes vasculogenic mimicry formation by vascular endothelial growth factor A mediating epithelial‐mesenchymal transition in salivary adenoid cystic carcinoma

**DOI:** 10.1111/cpr.12600

**Published:** 2019-04-03

**Authors:** Hao‐fan Wang, Sha‐sha Wang, Min Zheng, Lu‐ling Dai, Ke Wang, Xiao‐lei Gao, Ming‐xin Cao, Xiang‐hua Yu, Xin Pang, Mei Zhang, Jing‐biao Wu, Jia‐shun Wu, Xiao Yang, Ya‐jie Tang, Yu Chen, Ya‐ling Tang, Xin‐hua Liang

**Affiliations:** ^1^ State Key Laboratory of Oral Diseases, Department of Oral and Maxillofacial Surgery, National Clinical Research Center for Oral Diseases West China Hospital of Stomatology (Sichuan University) Chengdu Sichuan China; ^2^ Department of Stomatolog, Zhoushan Hospital Wenzhou Medical University Zhoushan China; ^3^ Key Laboratory of Fermentation Engineering (Ministry of Education), Hubei Provincial Cooperative Innovation Center of Industrial Fermentation, Hubei Key Laboratory of Industrial Microbiology Hubei University of Technology Wuhan China; ^4^ State Key Laboratory of Oral Diseases, Department of Oral Pathology, National Clinical Research Center for Oral Diseases West China Hospital of Stomatology (Sichuan University) Chengdu Sichuan China

**Keywords:** epithelial‐mesenchymal transition, hypoxia, salivary adenoid cystic carcinoma, vascular endothelial growth factor A, vasculogenic mimicry

## Abstract

**Objectives:**

To investigate the role of hypoxia in vasculogenic mimicry (VM) of salivary adenoid cystic carcinoma (SACC) and the underlying mechanism involved.

**Materials and methods:**

Firstly, wound healing, transwell invasion, immunofluorescence and tube formation assays were performed to measure the effect of hypoxia on migration, invasion, EMT and VM of SACC cells, respectively. Then, immunofluorescence and RT‐PCR were used to detect the effect of hypoxia on VE‐cadherin and VEGFA expression. And pro‐vasculogenic mimicry effect of VEGFA was investigated by confocal laser scanning microscopy and Western blot. Moreover, the levels of E‐cadherin, N‐cadherin, Vimentin, CD44 and ALDH1 were determined by Western blot and immunofluorescence in SACC cells treated by exogenous VEGFA or bevacizumab. Finally, CD31/ PAS staining was performed to observe VM and immunohistochemistry was used to determine the levels of VEGFA and HIF‐1α in 95 SACC patients. The relationships between VM and clinicopathological variables, VEGFA or HIF‐1α level were analysed.

**Results:**

Hypoxia promoted cell migration, invasion, EMT and VM formation, and enhanced VE‐cadherin and VEGFA expression in SACC cells. Further, exogenous VEGFA markedly increased the levels of N‐cadherin, Vimentin, CD44 and ALDH1, and inhibited the expression of E‐cadherin, while the VEGFA inhibitor reversed these changes. In addition, VM channels existed in 25 of 95 SACC samples, and there was a strong positive correlation between VM and clinic stage, distant metastases, VEGFA and HIF‐1α expression.

**Conclusions:**

VEGFA played an important role in hypoxia‐induced VM through regulating EMT and stemness, which may eventually fuel the migration and invasion of SACC.

## 1 INTRODUCTION

Salivary adenoid cystic carcinoma (SACC) is one of the most common malignant cancers of the salivary glands accounting for approximately 30% of all salivary malignant tumours.^1^, ^2^, ^3^ As an epithelial malignant tumour, SACC is characterized by early haematogenous dissemination, high incidence of lung metastasis and perineural spread.^4^, ^5^ The growth of SACC is quite slow and the 5‐year survival rates are 70%‐90%. However, the 5‐year survival rates of patients with distant metastasis are only 20%.^6^ Hence, it is necessary to address the underlying molecular mechanisms regulating metastatic dissemination of SACC.

Vasculogenic mimicry (VM), proposed by Maniotis in 1999, was defined as a type of vessel‐like structures lined with tumour cells without endothelial cells.^7^ VM had been found to evaluate in various aggressive cancers, including colorectal cancer,^8^ breast cancer,^9^ melanoma,^10^ and head and neck squamous cell carcinoma,^11^ suggesting that it was a novel hallmark of cancer. As newly defined mechanism to supply oxygen and nutrition to tumour cells, the high expression of VM was also viewed as a risk factor for poor prognosis, low survival, and invasion and metastasis in cancer patients.^12^ Recently, increasing evidence has showed that hypoxic microenvironment not only accelerated tumour invasion and metastasis, but also led to VM formation^13^, ^14^ and the expression of hypoxia‐inducible factor‐1α (HIF‐1α) was associated with VM in many cancers types, including breast cancer,^15^ ovarian cancer^16^ and colorectal cancer.^17^ Moreover, Ahluwalia et al reported that HIF‐1 by hypoxia condition adjusted vascular endothelial growth factor A (VEGFA) expression at the transcriptional level. VEGFA was a downstream target of HIF‐1, and angiogenesis produced by hypoxia was usually VEGFA‐dependent.^18^ And hypoxia induced EMT by regulating VEGFA. However, it is still not clear whether VEGFA is involved in the hypoxia microenvironment mediating VM formation.^19^, ^20^

In the previous study, our group has observed that CD133^+^ stem‐like SACC cells contributed to the migration and invasion of SACC through inducing VM formation.^21^ Here, we used a three‐dimensional culture model to detect VM formation in SACC cell lines under hypoxia condition and found that hypoxia contributed to the migration and invasion, epithelial‐mesenchymal transition (EMT) and VM formation of SACC cells. Overexpression of VEGFA boosted VM formation, the expression of VE‐cadherin, N‐cadherin, CD44 and ALDH1 in SACC cells. In addition, the presence of VM was positive association with the levels of VEGFA and HIF‐1 in SACC samples. These indicated that hypoxia may serve as an inducer of VM in SACC by targeting VEGFA‐mediated EMT.

## 2 MATERIALS AND METHODS

### 2.1 Ethics statement

All studies carried out on human specimens were approved by the Institutional Ethics Committee of the West China Medical Center, Sichuan University, China (No. WCHSIRB‐ST‐2012‐075). Every patient signed separate informed consent forms for sampling and molecular analysis.

### 2.2 Cell culture

The high metastatic potential cell line, SACC‐LM, and poor metastatic potential cell line (SACC‐83) were obtained from the State Key Laboratory of Stomatology, Sichuan University. All cell lines were maintained in high glucose DMEM (HyClone, USA) (10% foetal calf serum, HyClone）, 100 U/mL penicillin (Beyotime Biotechnology, China) and 0.1 mg/mL streptomycin (Beyotime Biotechnology) and incubated at 37°C at 5% CO_2_. Change the frequency of culture solution according to the growth rate and medium colour. Cells were passaged when cells covered 80%‐90% of the bottom.

### 2.3 Cell treatments

Cells were cultured in medium contained exogenous VEGFA (100 ng/mL, R&D, USA) and bevacizumab (inhibitor of VEGFA, 125 μg/mL, Selleck, USA), respectively.

### 2.4 Migration assays

Cells (2 × 10^5^/well) were seeded in 6‐well plates. When grow to 80%‐90% confluence, cells were scraped with 200 μL sterile micropipette tip to create a wound. The gap was photographed by inverted culture microscope (magnification, ×40). After 24 hours, the gap filling was photographed.

### 2.5 Invasion assay

Cell invasion was performed by BD BioCoat™ Matrigel™ Invasion Chamber (BD Biosciences, USA) according to the manufacturer's protocol. Briefly, cells (5 × 10^4^ cells) were plated on the top chamber in serum‐free DMEM and 10% FBS in DMEM in the bottom chamber. Cells were counted at a 100× magnification in standard microscopy 24 hours later.

### 2.6 Immunofluorescence

Cells were washed with 1× PBS for 3 minutes, fixed with 4% paraformaldehyde for 20 minutes and washed for three times with 1× PBS for 3 minutes. Then, cells were treated with 0.5% Triton X‐100 for 20 minutes at room temperature (this step was omitted when the antigen is expressed on the cell membrane) and washed with 1× PBS. Blocking was performed by normal goat serum for 30 minutes, followed by addition of primary antibody: rabbit anti‐E‐cadherin (1:400 dilutions, ProteinTech, China), anti‐N‐cadherin (1:300 dilutions, ProteinTech), rabbit anti‐Vimentin (1:200 dilutions, ProteinTech), rabbit anti‐VE‐cadherin (1:200 dilutions, ProteinTech) and rabbit anti‐VEGFA (1:200 dilutions, ProteinTech) to incubate overnight at 4°C, and then washed for three times with PBS. Add 1:200 fluorescent secondary antibody: goat anti‐Rabbit Alexa 488 (1:200 dilutions, ZSGB‐BIO, China), goat anti‐Rabbit Alexa 594 (1:200 dilutions, ZSGB‐BIO) and incubated at 37°C in dark for 2 hours. Add DAPI, incubated in dark for 5 minutes, washed with PBS for three times, and observed under a fluorescence microscope.

### 2.7 Vasculogenic mimicry assays

Wells of 24‐well plate were coated with Matrigel basement membrane matrix (BD). It was allowed to polymerase at room temperature for 1 hour and 37°C for 30 minutes. Cells were resuspended and seeded into a well at a density of 1 × 10^5^/mL, and then, the experimental group was incubated in a hypoxic incubator at 37°C, 94% N_2_, 5% CO_2_ and 1% O_2_, while the control group was cultured in a normoxic incubator at 37°C, 5% CO_2_ and 21% O_2_. Cell morphology and formation of cord‐like structures were observed using fluorescence inverted microscope (Olympus, Japan). Forty‐eight hours later, the length of tubes per field was quantified by counting in five randomly chosen 100× scopes. After 48 hours, the expression of VE‐cadherin and VEGFA was observed by immunofluorescence microscopy.

### 2.8 Scanning electron microscopy (SEM)

Cells cultured on a coverslip were rinsed with ice‐cold 1× PBS (pH 7.2) and fixed with 2% glutaraldehyde for 3 hours at 4°C. After washing with 1× PBS, the cells were fixed with 1% OsO4 for 2 hours and dehydrated in ethanol. The samples were then critical‐point dried and sputter‐coated with gold. Samples were examined, and images were acquired using a HITACHI S‐520 scanning electron microscope.

### 2.9 Confocal laser scanning microscopy (CLSM)

The cells were fixed in 4% paraformaldehyde (PFA) in 1× PBS at room temperature for 20 minutes. The cells were first pre‐treated and then blocked in 1× PBS with 0.5% Triton X‐100 and 5% BSA for 1 hour. Next, the samples were incubated with anti‐VEGFA (1:200 dilutions, ProteinTech) overnight at 4°C, followed by incubation with secondary antibodies goat anti‐Rabbit Alexa 488 (1:200 dilutions, ZSGB‐BIO). The samples were then analysed with a laser confocal microscope (OLYMPUS IX83, UltraVIEW VoX) with a 640 nm pulse. The images were collected with the Volivity software containing Acquisition, Quantitation and Visualization modules.

### 2.10 Flow cytometry (FCM)

The pre‐treated cells were harvested and washed twice with FCM buffer (PBS with 5% FBS and 0.1% NaN3). Cells were resuspended in PBS and incubated with PE‐anti‐human CD144 (BD Biosciences) for 30 minutes at 4°C.

### 2.11 Tumoursphere formation assay

For the tumoursphere formation assay, SACC‐LM cells were seeded at a density of 800 cells/ mL of tumoursphere formation medium in ultra‐low attachment 12‐well plates for 12 days. The tumourspheres formed (spherical, non‐adherent cell‐masses  >90 μm in diameter) were photographed and counted under inverted phase contrast microscope.

### 2.12 Western‐blot

The isolated cells were lysed in lysis buffer. The protein concentration was then determined. Equal amounts of protein were analysed by SDS‐PAGE, followed by electrophoretic transfer to PVDF membranes (Millipore Corp., Billerica, MA, USA). The membrane was blocked for 1 hour with 5 mL blocking buffer (5% skim milk in TBST) and incubated overnight at 4°C with 5% skim milk diluted primary antibody: Rabbit anti‐Human VE‐cadherin antibody (1:500 dilutions, ProteinTech); Rabbit anti‐Human VEGFA antibody (1:500 dilutions, ProteinTech); and Rabbit anti‐Human GAPDH antibody (1:500 dilutions, ProteinTech). The next day, the membranes were washed three times with TBST and incubated with 2% skim milk diluted secondary antibody for 2 hour. After washing, the immunoreactive protein bands were visualized using the BIO‐RAD gel imaging system. GAPDH was used as an internal control.

### 2.13 Real‐Time PCR (RT‐PCR)

Total RNA was extracted from cells using Trizol (Invitrogen, USA). Then, we synthesized cDNA using a reverse transcription kit (TaKaRa, China) following the manufacturer's instructions. Primer sequences used for PCR were as follows: VE‐cadherin: F: ATGAGATCGTGGTGGAAGCG R: TGTGTACTTGGTCTG GGTGA; VEGFA: F: CAGGCTGCTGTAACGATGAA R: TTTCTTGCGCTTTCGTTTTT; Vimentin: F: ACC AAG ACA CTA TTG GCC GCC T R: CCC TCA GGT TCA GGG AGG AAA AGT; E‐Cadherin: F: ATTGCTCACATTTCCCAACTC R: GTCACCTTCAGCCATCCT; N‐cadherin: F: TGCGGTACAGTGTAACTGGG R: GAAACCGGGCTATCTGCTCG; Twist1: F: TGTCCGCGTCCCACTAGC R: TGTCCATTTTCTCCTTCTCTGG; Snail: F: GACTACCGCTGCTCCATTCCA R: TCCTCTTCATCACTAATGGGGCTTT; Slug1: F: AGATGCATATTCGGACCCAC R: CCTCATGTTTGTGCAGGAGA; VEGFR1: F: ACCTCCGTGCATGTGTATGA R: TGGTGCATGGTTCTGTTGTT; VEGFR2: R: CCGTCAAGGGAAAGACTACG R: AGATGCTCCAAGGTCAGGAA; GAPDH: F: TGGCCAAGGTCATCCATGAC R: TGTCATACCAGGAAATGAGCTTG.

### 2.14 Immunohistochemical (IHC) and histochemical double‐staining methods

Specimens of surgical tumour tissues from SACC patients were fixed with 4% formalin, paraffin embedded and sectioned (4 μm). The tissue sections were then deparaffinized and dehydrated followed by incubation in 3% hydrogen peroxide for 10 minutes. Slides were stained with primary antibodies at 4°C overnight after blocking with appropriate serum. Corresponding secondary antibodies were used for 1h at room temperature. Targeted molecules were detected following DAB staining for immunohistochemistry. Tissues were sectioned for CD31 immunohistochemical staining, followed by PAS staining. Slides were finally counterstained with haematoxylin. Two independent investigators blinded to sample identify the anti‐VEGFA antibody (1:200 dilutions, ProteinTech) and anti‐CD31 antibody (1:100 dilutions, ProteinTech) primary antibody, respectively. PBS was used as the primary antibody for the negative controls.

### 2.15 Statistical analysis

The data were recorded as mean ± SD (standard deviation) and evaluated by SPSS 21.0. Differences between groups were analysed by Student's *t* test or chi‐squared test. *P* < 0.05 was considered to indicate a statistically significant result.

## 3 RESULTS

### 3.1 Hypoxia contributed to migration and invasion, and EMT of SACC cells

**Figure 1:**
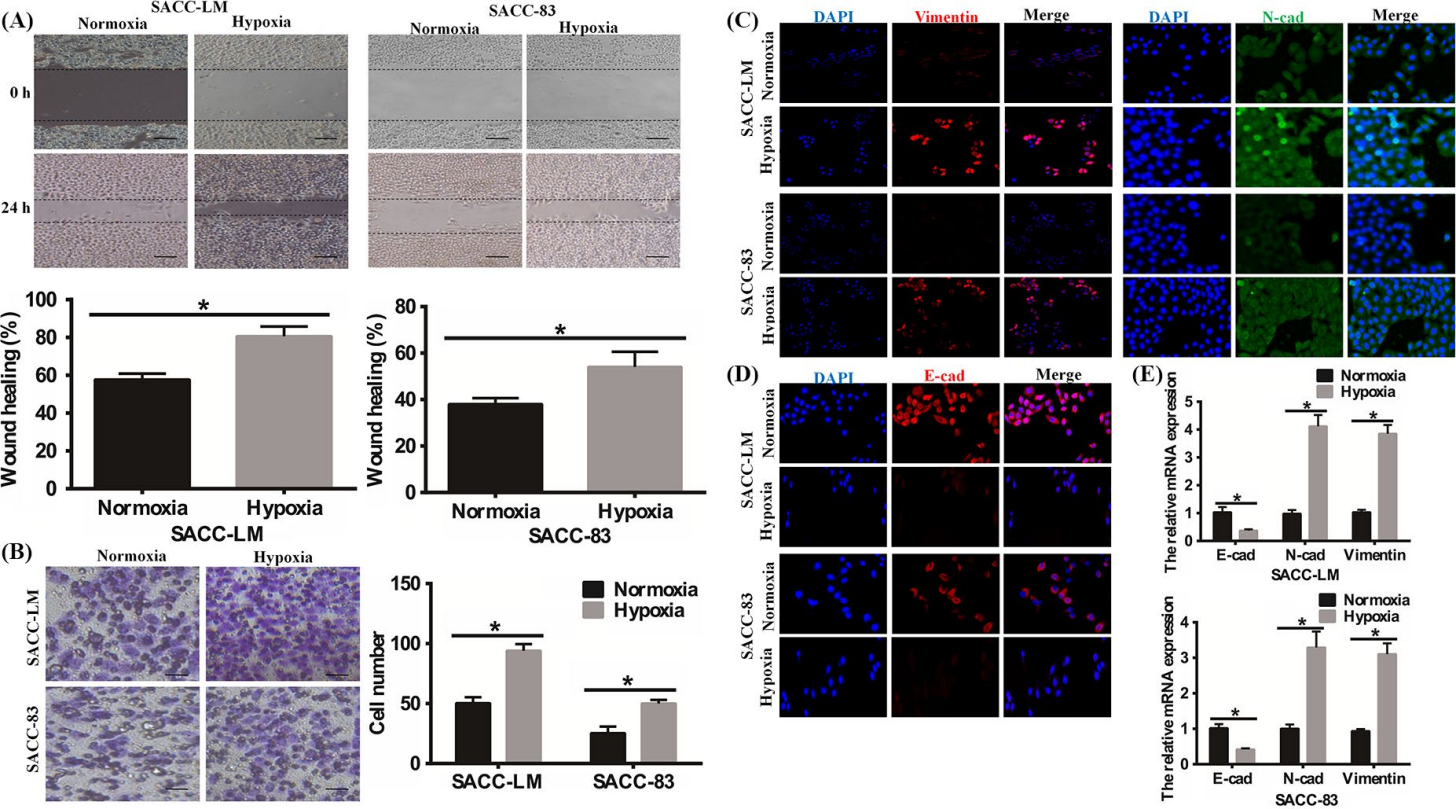
Changes in the capacity of invasion and metastasis of the SACC cells. A, Wound healing assays (magnification, ×40; bars, 200 μm) revealed that hypoxia promoted cells migration of SACC‐LM and SACC‐83. The results were statistically significant (*P < 0.05). B, Number of invasion cells was assessed in the invasion assay (magnification, ×100; bars, 100 μm). Results showed that hypoxia enhanced SACC cells invasion (*P < 0.05). C, The immunofluorescence staining showed that hypoxia up‐regulated the expression of N‐cadherin and Vimentin (magnification, ×100). D, The immunofluorescence staining showed that hypoxia down‐regulated the level of E‐cadherin (magnification, ×100; bars, 100 μm). E, The RT‐PCR showed the changes of E‐cadherin, N‐cadherin and Vimentin in mRNA level

To investigate the role of hypoxia in SACC, we applied wound healing and transwell invasion assays to observe migration and invasive abilities under hypoxia. As shown in Figure 1A,B, hypoxia dramatically increased the migratory and invasive behaviours of SACC‐83 and SACC‐LM cells. Then, we further detected the relationship between hypoxia and EMT and found that SACC cells exhibited a significant up‐regulation of mesenchymal markers, N‐cadherin and Vimentin (Figure 1C), meanwhile dramatically down‐regulation of E‐cadherin (Figure 1D) by immunofluorescence. Real‐time PCR analyses also revealed that the mRNA expression of E‐cadherin decreased, as well as N‐cadherin and Vimentin mRNAs increased in SACC‐83 and SACC‐LM cells under hypoxia (Figure 1E). Besides, the expression of EMT associated transcription factors, Twist1, Snail, and Slug1 also up‐regulated under hypoxia in SACC cells (Figure S1A). These suggested that hypoxia promoted migration and invasion, and EMT of SACC cells.

### 3.2 Hypoxia promoted VM formation in SACC cells

**Figure 2:**
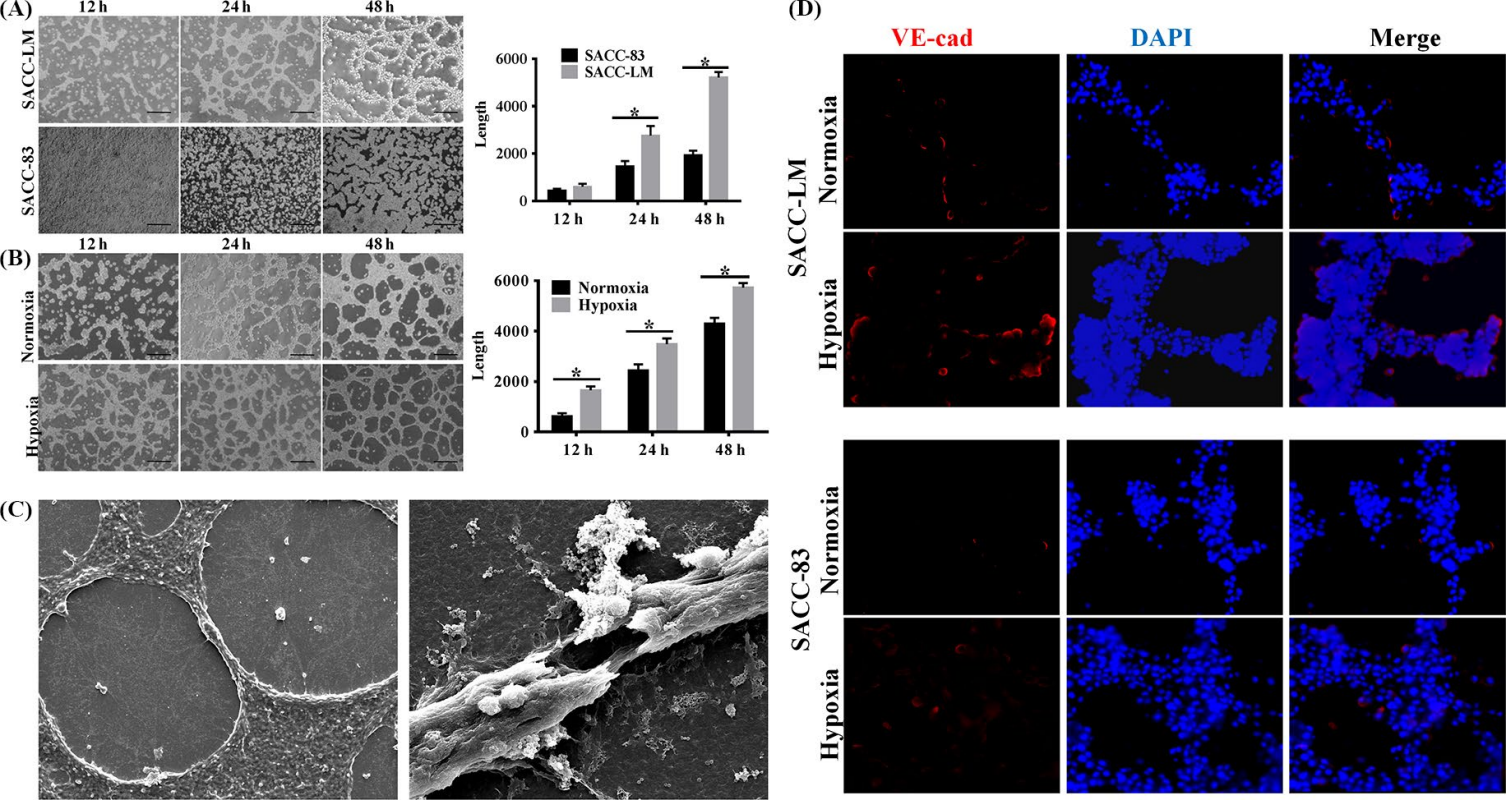
Effect of hypoxia on VM formation in SACC cell lines. A, Tube‐like structure formation on Matrigel in SACC‐LM and SACC‐83 cells under normoxia. The SACC‐LM showed a stronger ability of VM formation. For quantitative analysis, six nonoverlapping fields were selected from each culture well and the experiment was performed in quadruplicate (*P < 0.05). B, Hypoxia promoted the VM formation of SACC cells. For quantitative analysis, six nonoverlapping fields were selected from each culture well and the experiment was performed in quadruplicate. (*P < 0.05). C, Scanning electron microscopy was performed to show the tubular profiles were hollow and lined by flattened cancer cells after 7 days on the three‐dimensional cultures. The panel on the left was representative image of cross section of tubular structure. The panel on the right was representative image of vertical sectiono. D, The immunofluorescence staining showed that hypoxia promoted VE‐cadherin of SACC cells

Then, we plated SACC cell lines, SACC‐83 and SACC‐LM, on the surface of Matrigel to establish the 3D culture model, and assessed the ability of VM formation under hypoxia during 48 hours. Our data showed that SACC‐LM cells with high metastatic potential exhibited the formation of channel‐like structures after 12 hours incubation on matrigel in normoxia or hypoxia group. After 48 hours, we observed interconnected loops and networks in SACC‐LM cells in both normoxia and hypoxia groups. In contrast, there were less tubular or sinusoidal channels in poor metastatic SACC‐83 cells (Figure 2A). For the two cell lines, quantification of the length of channel‐like structures showed that there was a significant increase after 48 hours incubation in hypoxia in comparison with normoxia condition (*P < *0.05, Figure 2B). To further define the structure of the tubular networks, scanning electron microscopy was performed on the three‐dimensional cultures. We found that tubular profiles were hollow and lined by flattened cancer cells after 7 days (Figure 2C). Furthermore, immunofluorescence staining revealed that the expression of VE‐cadherin, marker of VM, enhanced in SACC‐LM under hypoxia, compared to normoxia. However, there was no statistical difference between the normoxia and hypoxia group in SACC‐83 cells (Figure 2D). Besides, the mRNA levels of VEGFR1 and VEGFR2, markers of VM, remarkably enhanced under hypoxia (Figure S1B). These results suggested that the vital role of hypoxic condition in the formation of vasculogenic‐like networks and metastatic potential was positive correlation with the ability of VM formation in SACC in vitro.

### 3.3 VEGFA was crucial for hypoxia‐induced VM of SACC cells in vitro

**Figure 3:**
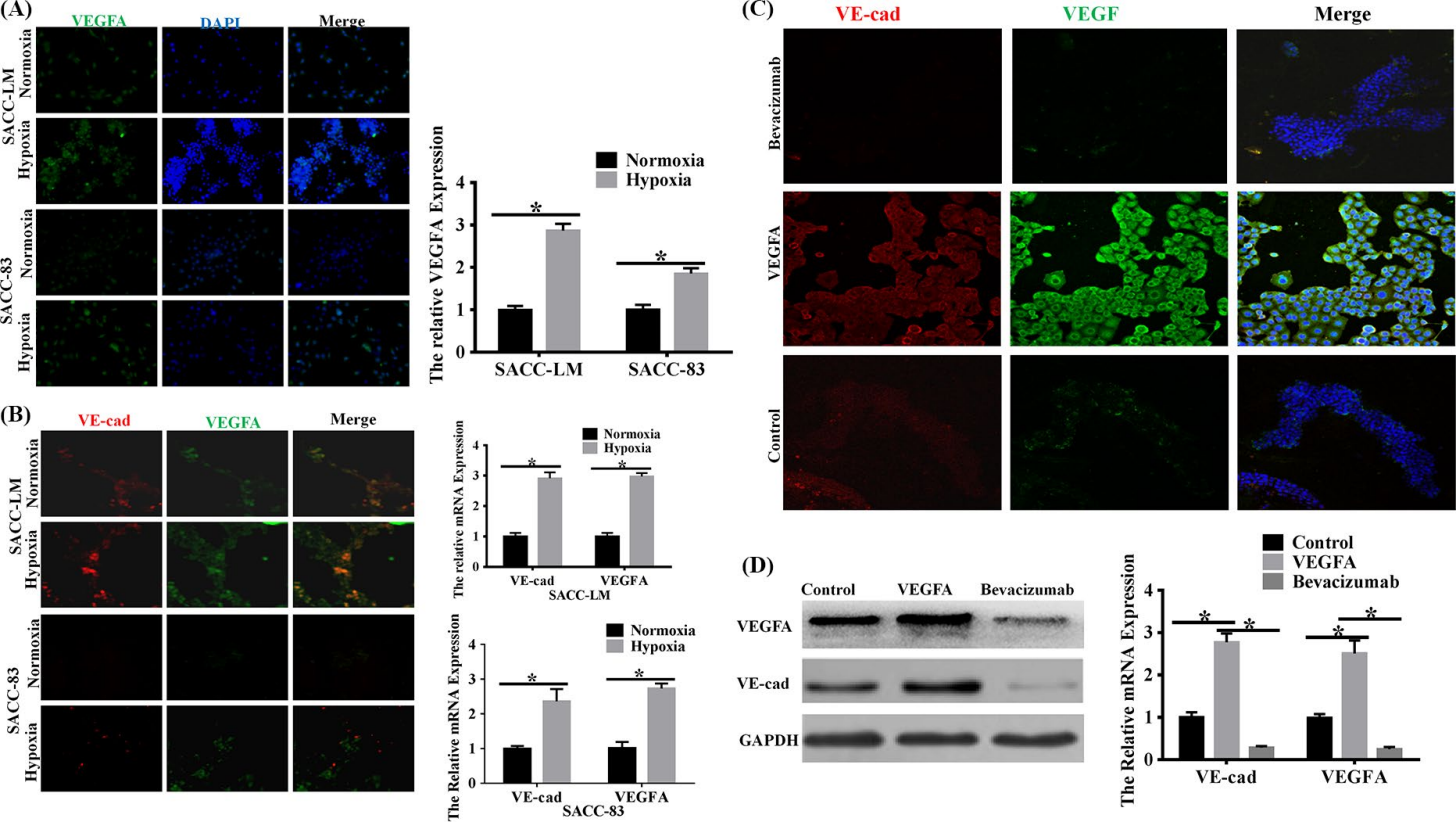
VEGFA was involved in VM formation of SACC‐LM and SACC‐83 cells in vitro. A, Immunofluorescence staining and RT‐PCR assessed the effect of hypoxia on VEGFA protein and mRNA expression in SACC cell lines, respectively. The data showed that hypoxia enhanced the level of VEGFA of SACC cells in 2D culture in both protein and mRNA levels (magnification, ×100; bars, 100 μm, *P < 0.05). B, VEGFA expression in SACC‐LM and SACC‐83 cells was assessed by immunofluorescence staining and RT‐PCR. The data showed that VEGFA was overexpression accompanied by high expression of VE‐cadherin under hypoxia (magnification, ×100; *P < 0.05). C, CLSM (magnification, ×200) was performed to assess the role of VEGFA on VE‐cadherin of SACC‐LM cells. The data showed that VEGFA enhanced the level of VE‐cadherin while bevacizumab suppressed its expression. D, Western blot was performed to assess the role of VEGFA on VE‐cadherin of SACC‐LM cells. The data showed that VEGFA enhanced the level of VE‐cadherin while bevacizumab suppressed its expression. E, RT‐PCR (*P < 0.05) was performed to assess the role of VEGFA on VE‐cadherin of SACC‐LM cells. The data showed that VEGFA enhanced the level of VE‐cadherin while bevacizumab suppressed its expression

As we known, VEGFA plays a key role in tumour‐associated angiogenesis, but its role in vasculogenic mimicry remains unclear. In order to answer this question, we first compared VEGFA expression in SACC‐83 and SACC‐LM cells by immunofluorescence and RT‐PCR. Our results demonstrated that VEGFA expression was elevated in SACC‐LM owning higher VM‐forming potential compared to SACC‐83 cells owning poor VM‐forming potential, especially under hypoxia condition (Figure 3A). Then, tube formation assay was conducted by seeding cells on Matrigel and we assessed the level of VEGFA in VM‐forming SACC cells. We found that the expression of VEGFA remarkably increased in tube‐forming cells under hypoxic niche, especially in SACC‐LM (Figure 3B). It hinted that VEGFA was associated with VM formation in hypoxia. So we next treated SACC‐LM cells with exogenous VEGFA or bevacizumab under hypoxia. After 6 hour incubation on matrigel, compared to the control group or VEGFA‐inhibition group, the SACC‐LM cells treated with VEGFA showed morphological changes and tendency to form VM‐structures. After 48 hours, both the VEGFA‐stimulated group and control group formed typical tube‐like structures, while there were just a few such structures in VEGFA‐inhibited group (Figure 3C). Subsequently, we investigated the effect of VEGFA on the level of VE‐cadherin in hypoxic microenvironment. Compared to the control group, exogenous VEGFA could enhance the mRNA and protein levels of VE‐cadherin under hypoxia assessed by RT‐PCR and Western blot, respectively. Contrarily, bevacizumab declined the expression of VE‐cadherin (Figure 3D). The expression of VE‐cadherin was in accordance with the level of VEGFA. These data indicated a possible role of VEGFA in regulating the hypoxia‐induced tube formation capability of SACC in vitro.

### 3.4 VEGFA resulted in hypoxia‐induced VM formation by inducing EMT in SACC

**Figure 4:**
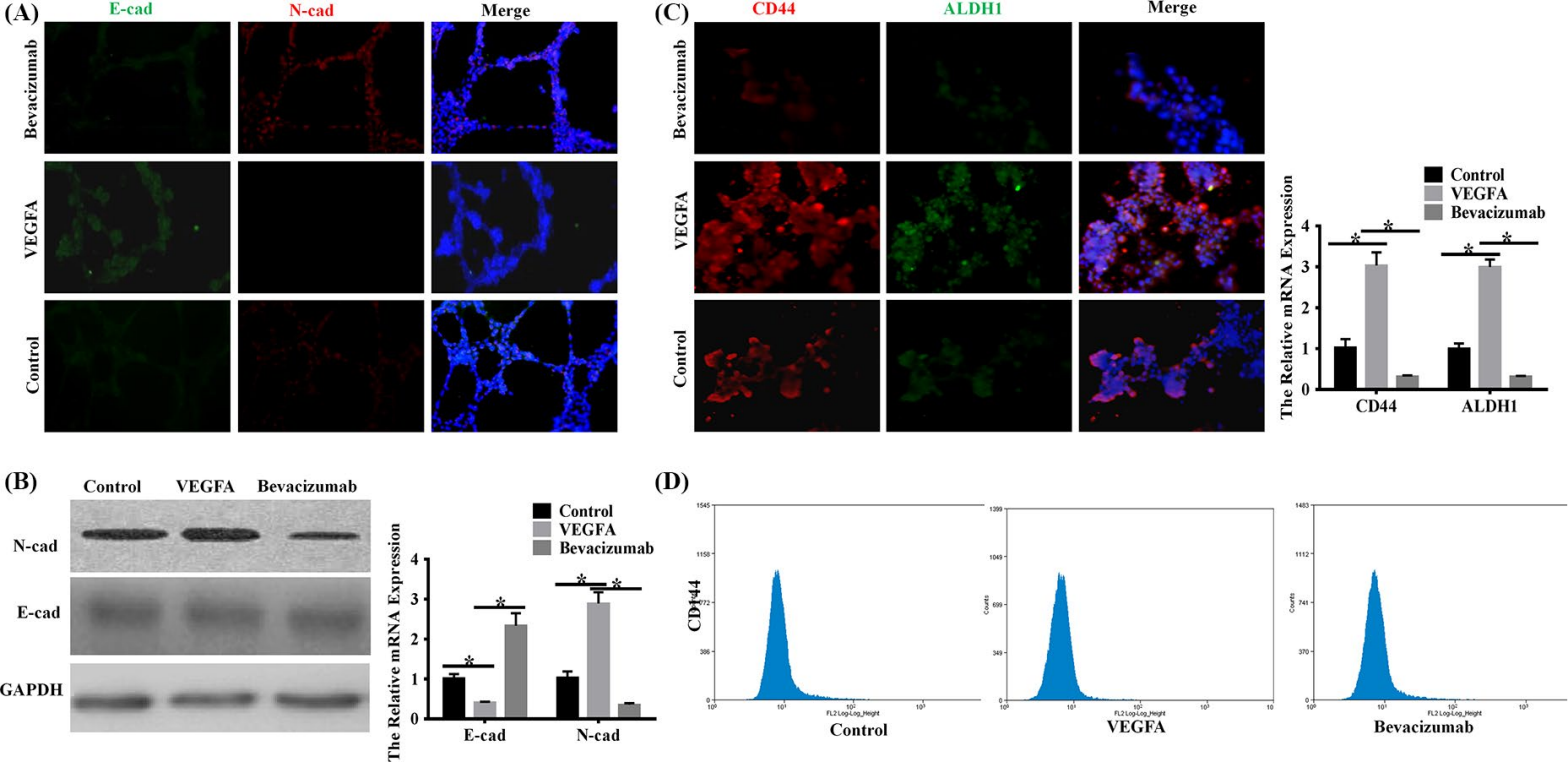
Exogenous VEGFA promoted EMT and stemness of SACC cells in vitro. A, Immunofluorescence staining (magnification, ×100) was performed to assess the role of VEGFA on EMT of SACC‐LM cells. The data showed that VEGFA enhanced the level of N‐cadherin and inhibition the expression of E‐cadherin while bevacizumab resulted in reverse changes. B, Western blot and RT‐PCR (*P < 0.05) were performed to assess the role of VEGFA on EMT of SACC‐LM cells. The data showed that VEGFA enhanced the level of N‐cadherin and inhibition the expression of E‐cadherin while bevacizumab resulted in reverse changes. C, Immunofluorescence staining (magnification, ×100; bars, 100 μm) and RT‐PCR (*P < 0.05) were performed to assess the role of VEGFA on stemness of SACC‐LM cells. The data showed that VEGFA enhanced the levels of CD44 and ALDH1 while bevacizumab resulted in reverse changes. D, Flow cytometry showed that the expression of CD144 had no difference among three groups

For epithelial tumour cells, the capability of mimicking endothelial functions played an important role in VM formation.^22^ Thus, we speculated that VEGFA contributed to hypoxia‐mediated VM‐forming though inducing EMT. Our results showed that overexpressed VEGFA in SACC‐LM led to remarkably increased N‐cadherin expression while E‐cadherin was almost undetectable in the hypoxia‐stimulated tube structures in both mRNA and protein levels after 48 hours seeded on Matrigel. The inhibition of VEGFA was associated with a marked decrease in N‐cadherin and unchanged level in E‐cadherin (Figure 4A,B). It was documented that EMT endowed epithelial cancer cells with the self‐renewal capacity.^23^ And our group previously proved that CD133^+^, the stemness marker, was positively associated with VM formation in SACC specimens.^21^ Therefore, we inferred that VEGFA also contributed to the acquisition of stem cell phenotype in SACC cells. Immunofluorescence staining revealed that compared to the control group, SACC‐LM treated with exogenous VEGFA showed the increase of stemness marker‐CD44 and ALDH1 expression in VM‐forming cells. On the contrary, bevacizumab led to a significant decrease in CD44 and ALDH1 expression of tube‐forming cells (Figure 4C). However, the flow cytometry showed that the expression of CD144, another stemness marker, had no difference among the three groups (Figure 4C). We assessed the sphere formation of SACC‐LM under the stimulation of exogenous VEGFA or bevacizumab. And we observed that the exogenous VEGFA heightened the sphere‐forming capacity while bevacizumab inhibited the tumoursphere formation (Figure S1C). Collectively, these experiments suggested that VEGFA promoted hypoxia‐induced VM formation, which may be mediated by the EMT process and cancer stem cells (CSCs) in SACC cells in vitro.

### 3.5 Presence of VM in SACC tissues correlated with high expressions of VEGFA and HIF‐1α

**Figure 5:**
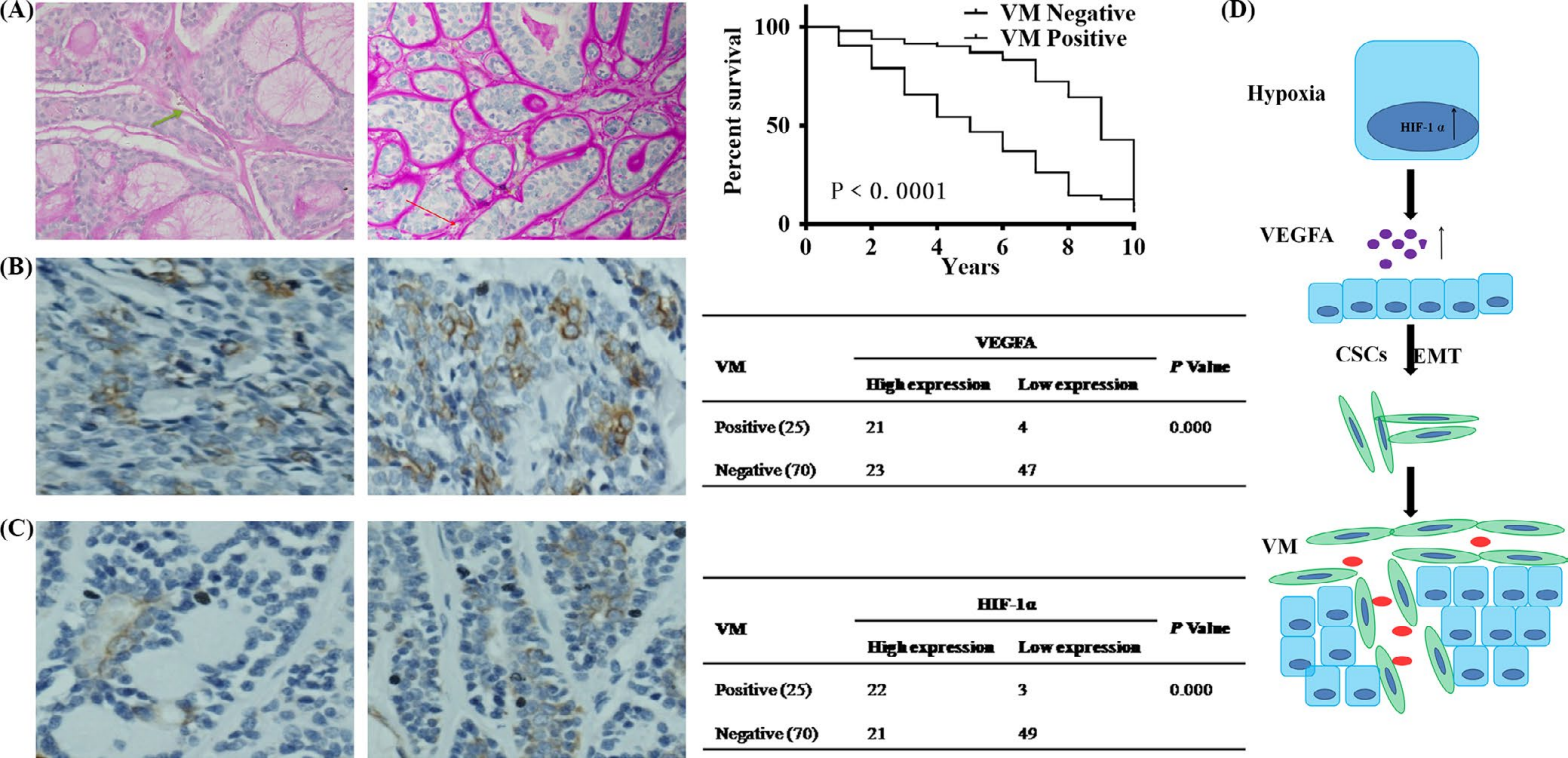
VM formation correlated with VEGFA and HIF‐1α expression in SACC tissues. A, CD31/PAS double staining was utilized to access the presence of VM. The panel on the left showed endothelial cell‐dependent blood vessels which were CD31^+^PAS^−^ channels (green arrow). The panel on the right showed VM which was CD31^−^PAS^+^ (red arrow) (magnification, ×100). B, VEGFA in SACC showed mainly cytoplasmic staining (magnification, ×100). Different expression levels of VEGFA in VM‐positive and VM‐negative tumours from the two distinct SACC patients, and the evaluation of the relationship between VEGFA expression and VM formation in SACC tissues using *χ* ^2^ tests. C, HIF‐1α in SACC showed mainly cytoplasmic and nuclear staining (magnification, ×100). Different expression levels of HIF‐1α in VM‐positive and VM‐negative tumours from the two distinct SACC patients, and the evaluation of the relationship between HIF‐1α expression and VM formation in SACC tissues using *χ* ^2^ tests. D, Schematic representation of the role of VEGFA in VM formation of SACC

The presence of VM was authenticated by the presence of PAS‐positive loops and/or contained red blood cells that were negative for the endothelial cell marker CD31, while the endothelial vascular channels were positive for CD31 staining (Figure 5A). As shown in Table 1, 25 tissue samples from 95 cases of SACC (26.3%) were VM positive. In addition, there was a significant association between the presence of VM and the clinic stage (*P* = 0.006) and distant metastases (*P* = 0.000), but there was no association between VM and patients’ age, gender, position and pathological subtypes (*P* > 0.05). As shown in Figure 5B, high levels of expression of VEGFA was present in 47 of the 95 cases (49.47%) and in 18 of the 25 VM‐positive samples (72.0%) but in only 29 of the 70 VM‐negative samples (41.43%). VEGFA staining was mainly cytoplasmic. Statistically, there was a significant correlation between high expression of VEGFA and presence of VM (*r* = 0.412, *P* = 0.000) (Figure 5B). The positive staining of HIF‐1α was located in the cytoplasm and the nuclei. Statistically, there was a significant correlation between high expression of HIF‐1α and presence of VM (*r* = 0.457, *P* = 0.000; Figure 5C). Taken together, the results show a positive association of VM and VEGFA or HIF‐1α in SACC.

## 4 DISCUSSION

Hypoxia, a common characteristic of solid tumours, has been reported to activate migration, invasion and VM formation in several tumour models including melanoma,^23^ oral squamous cell carcinoma^24^ and glioma.^25^ Here, we found that hypoxia accelerated VM formation of SACC and VEGFA was a critical downstream molecule that mediated the hypoxia‐controlled SACC VM in vitro. And we identified that VEGFA regulated the cell plasticity and stemness to promote VM formation of SACC cells. In addition, the high expression of HIF‐1α and VEGFA obviously associated with the VM density in SACC samples. Thus, the current study linked VEGFA and consequential activation of EMT and stemness with VM formation in SACC and indicated the potential of VEGFA to act as therapeutic targets in SACC.

Here, we found that the migration and invasion abilities of SACC cells under hypoxic condition were stronger than under normoxia in vitro*, *and hypoxia induced EMT of SACC cells. In line with our study, hypoxic microenvironment could accelerate the migration and invasion of both MDA‐MB‐231 cells and MCF‐7 cells.^26^ Kim et al^27^ illustrated that hypoxic condition augmented the aggressive phenotypes including mobility and invasiveness of human malignant mesothelioma cells in vitro. Lin et al^28^ found that hypoxia induced the EMT and facilitated cancer dissemination in non‐small cell lung cancer cells. Then, the data of the 3D culture conformed that hypoxic condition significantly promoted the formation of tube‐like structures and the expression of VE‐cadherin compared to normoxia in SACC cells, and the presence of VM was positively related to the high expression of HIF‐1α. In agreement with our results, Du *et al* 16 found that hypoxia increased VM formation of ovarian cancer cell lines SKOV3 and OVCAR3 in vitro. Wang et al^14^ demonstrated that the high expression of HIF‐1α was positive correlation with VM in 201 HCC sample tissues. Salinas‐Vera et al^13^ observed a dramatic increase in VM‐related structures when triple negative breast cancer cell lines MDA‐MB‐231 and Hs‐578T cells were grown after 12 hour hypoxic incubation on Matrigel. These indicated that hypoxia promoted the aggressiveness of SACC cells.

A number of previous studies have demonstrated that hypoxia closely associated with angiogenesis via VEGFA^29^, ^30^; however, it is unclear whether VEGFA has equally important significance in hypoxia‐induced VM in SACC. Our results showed that VEGFA was remarkably increased in SACC cells under hypoxia condition. Exogenous VEGFA strengthened the capability of forming vessel‐like structures and VE‐cadherin expression, while bevacizumab obviously restrained VM formation and VE‐cadherin expression. Moreover, the high level of VEGFA showed a significant correlation with the VM in SACC tissues. The data were supported by the report of Vartanian et al,^31^ who showed that VEGFA was essential for the initiation of melanoma cells into capillary‐like structure in vitro. The knockdown of VEGFR1 completely prevented Matrigel‐induced VM formation. Hence, VEGFA/VEGFR1 pathway was considered to contribute to the formation of VM in melanoma. Azad et al^32^ confirmed that VEGF/VEGFR promoted VM in MCF10 cells via Hippo pathway. These highlighted the role of VEGFA in promoting hypoxia‐mediated VM in SACC. However, Xu et al^33^ found that short‐term anti‐VEGFA treatment induced increase VM and metastasis in SKOV3^LUC+^ tumour‐bearing mice. Schnegg et al^34^ found that tumour xenografts originated from sensitive to VEGFA inhibition underwent an adaptation *via *HIF‐1 expression and an increase in CD144^+^ VM, while tumour xenografts of cell populations resistant to this inhibition did not exhibit these features compared to the control counterparts. Thus, VEGFA may perform different roles in the VM in the different tumour cell types and much should be done.

EMT, the reversible dedifferentiation process of polarized epithelial cells, has been shown to play an important role in acquiring endothelial‐like properties to form vessel‐like structures in epithelial cancers.^35^ Meng et al^36^ found that Hsp90β enhanced the VE‐cadherin promoter activity by interacting with EMT‐related transcription factor Twist1 in hepatocellular carcinoma. Similarly, the high level of ZEB1, another EMT‐related transcription factor, was strongly associated with the presence of VM in 96 prostate cancer samples (*P* < 0.05).^37^ On the other hand, evidence confirmed that VEGFA stimulation of normal epithelial cells and differentiated carcinoma cells could induce an EMT. VEGFA could stimulate EMT of pancreatic neuroendocrine tumour cells in vitro.^38^ In breast cancer, VEGFA led to EMT by up‐regulating SOX2 expression in vivo and in vitro*.* 39 VEGFA could sustain EMT in sonic hedgehog medulloblastoma stem cells via Nrp2.^40^ Hence, we proposed a hypothesis that VEGFA may accelerate hypoxia‐induced VM by affecting EMT in SACC. In order to verify this, we examined the role of VEGFA in hypoxia‐controlled VM formation in SACC. The data showed that exogenous VEGFA enhanced the expression of N‐cadherin and sharply suppressed the expression of E‐cadherin in channel‐like structures. In the contrary, inhibition of VEGFA made N‐cadherin down‐regulate accompanied by unchanged E‐cadherin level. These results strongly suggested that VEGFA‐induced EMT may a critical step in hypoxia‐caused VM in SACC. Furthermore, EMT is considered to be sufficient to endow and maintain cancer cells with stemness. There are remarkable similarities between cancer stem cells (CSCs) and cancer cells with mesenchymal phenotype, both of which are highly invasive, resistant against common anticancer treatment and thought to cause metastatic growth. And the two phenotypes are reversible and can be interchanged via EMT or CSC phenotype interconversion.^41^, ^42^ A growing body of evidence suggests that CSCs are also involved in regulating VM formation.^43^ Hence, we assessed the effect of VEGFA on cancer cells stemness and found that overexpression of VEGFA accelerated stemness marker CD44 and ALDH1 expression in VM‐forming SACC cells under hypoxia, while suppression of VEGFA reduced CD44 and ALDH1 expression. Similarly, Jang et al^44^ found that VEGFA up‐regulated Bmi1, tumourspheres and ALDH1 activity in primary human ovarian cancer cell lines via VEGFR2‐dependent Src activation.^44^ Ramezani et al^45^ demonstrated that human glioblastoma stem‐like cells promoted themselves proliferation by secreting the VEGFA in an autocrine manner. Therefore, we inferred that VEGFA may affect VM by endowing SACC cells stemness.

In addition, we detected PAS^+^/CD31^−^ vessel‐like structure contained red blood cells in 25 of 95 SACC samples and the data showed that the VM positively correlated with advanced clinic stage and distance metastasis of SACC patients. Wang et al^46^ showed that VM was detected in a total of 25 (27.2%) of 92 prostate cancer patients whose presence was positive correlation with advanced TNM stage (*P* = 0.001), and the presence of lymph node (*P* = 0.001) as well as distant metastases (*P* = 0.025). Williamson et al^47^ pointed out that higher levels of VM were associated with worse 3‐year survival in 41 limited‐stage small cell lung cancer patients’ biopsies (*P* < 0.025). You et al^48^ demonstrated that VM was existent in 29/127 (22.8%) primary gastric cancer cases, and the presence of VM was associated with tumour size, differentiation, depth of tumour invasion, stage and lymph node metastases. The data confirmed that VM might be a poor prognosis marker of human carcinoma.

Taken together, in present study, we confirmed that hypoxia accelerated the VM of SACC cells, and VEGFA was a critical downstream molecule that mediated the hypoxia‐controlled EMT inducing VM in SACC. In human SACC tissues, the presence of VM was positive correlation with the advanced clinic stage and distance metastasis, as well as the levels of VEGFA and HIF‐1α in SACC tissue. These indicated that hypoxia may serve as an inducer of VM in SACC by targeting VEGFA‐mediated EMT, which provided a new sight in the search the therapeutic target of SACC.

## CONFLICT OF INTEREST

The authors confirm that there are no conflicts of interest.

## AUTHOR CONTRIBUTION

Study design: YLT, XHL; Data Collection: HFW, SSW, MZ, LLD, KW, XLG; Data analysis: MXC, XHY, XP, MZ, JBW; Manuscript preparation: JSW, XY, YJT, YC.

5

**Table 1:** Clinicopathological features of SACC patients and their association with VM expression

Clinicopathological features	*n*	VM		*P* value
		Positive (n = 25)	Negative (n = 70)	
Age				
<45	38	9	29	0.635
≥45	57	16	41	
Gender				
Male	42	15	27	0.640
Female	53	10	43	
Site				
Major salivary gland	70	16	54	0.200
Minor salivary gland	25	9	16	
Clinic stage				
I/II	45	9	36	0.006
III/IV	50	19	31	
Histological subtype				
Tubular/cribriform	58	39	19	0.196
Solid	37	20	17	
Distant metastasis				
Yes	35	30	5	0.000
No	60	28	32	

## References

[1] Tian Z , Li L , Wang L , Hu Y , Li J . Salivary gland neoplasms in oral and maxillofacial regions: a 23‐year retrospective study of 6982 cases in an eastern Chinese population. Int J Oral Maxillofac Surg. 2010;39:235‐242. 19951834 10.1016/j.ijom.2009.10.016

[2] Wang Y , Zhang CY , Xia RH , et al. The MYB/miR‐130a/NDRG2 axis modulates tumor proliferation and metastatic potential in salivary adenoid cystic carcinoma. Cell Death Dis. 2018;9:917. 30206227 10.1038/s41419-018-0966-2 PMC6134089

[3] Zhang J , Peng B , Chen X . Expressions of nuclear factor kappaB, inducible nitric oxide synthase, and vascular endothelial growth factor in adenoid cystic carcinoma of salivary glands: correlations with the angiogenesis and clinical outcome. Clin Cancer Res. 2005;11:7334‐7343. 16243805 10.1158/1078-0432.CCR-05-0241

[4] Yao X , Wang Y , Duan Y , et al. IGFBP2 promotes salivary adenoid cystic carcinoma metastasis by activating the NF‐κB/ZEB1 signaling pathway. Cancer Lett. 2018;432:38‐46. 29885520 10.1016/j.canlet.2018.06.008

[5] Weng LX , Wang GH , Yao H , Yu MF , Lin J . Epigallocatechin gallate inhibits the growth of salivary adenoid cystic carcinoma cells via the EGFR/Erk signal transduction pathway and the mitochondria apoptosis pathway. Neoplasma. 2017;64:563‐570. 28485162 10.4149/neo_2017_410

[6] Adams A , Warner K , Nör JE . Salivary gland cancer stem cells. Oral Oncol. 2013;49:845‐853. 23810400 10.1016/j.oraloncology.2013.05.013 PMC3744607

[7] Maniotis AJ , Folberg R , Hess A , et al. Vascular channel formation by human melanoma cells in vivo and in vitro: vasculogenic mimicry. Am J Pathol. 1999;155:739‐752. 10487832 10.1016/S0002-9440(10)65173-5 PMC1866899

[8] Zhang S , Fu Z , Wei J , Guo J , Liu M , Du K . Peroxiredoxin 2 is involved in vasculogenic mimicry formation by targeting VEGFR2 activation in colorectal cancer. Med Oncol. 2015;32:414. 25471788 10.1007/s12032-014-0414-9

[9] Tu DG , Yu Y , Lee CH , et al. Hinokitiol inhibits vasculogenic mimicry activity of breast cancer stem/progenitor cells through proteasome‐mediated degradation of epidermal growth factor receptor. Oncol Lett. 2016;11:2934‐2940. 27073579 10.3892/ol.2016.4300 PMC4812586

[10] Ruffini F , Graziani G , Levati L , Tentori L , D'Atri S , Lacal PM . Cilengitide downmodulates invasiveness and vasculogenic mimicry of neuropilin 1 expressing melanoma cells through the inhibition of αvβ5 integrin. Int J Cancer. 2015;136:E545‐558. 25284767 10.1002/ijc.29252

[11] Wang W , Lin P , Sun B , et al. Epithelial‐mesenchymal transition regulated by EphA2 contributes to vasculogenic mimicry formation of head and neck squamous cell carcinoma. Biomed Res Int. 2014;2014:803914. 24864260 10.1155/2014/803914 PMC4016880

[12] Bai J , Yeh S , Qiu X , et al. TR4 nuclear receptor promotes clear cell renal cell carcinoma (ccRCC) vasculogenic mimicry (VM) formation and metastasis via altering the miR490‐3p/vimentin signals. Oncogene. 2018;37:5901‐5912. 29973687 10.1038/s41388-018-0269-1

[13] Salinas‐Vera YM , Marchat LA , García‐Vázquez R , et al. Cooperative multi‐targeting of signaling networks by angiomiR‐204 inhibits vasculogenic mimicry in breast cancer cells. Cancer Lett. 2018;432:17‐27. 29885516 10.1016/j.canlet.2018.06.003

[14] Wang M , Zhao X , Zhu D , et al. HIF‐1α promoted vasculogenic mimicry formation in hepatocellular carcinoma through LOXL2 up‐regulation in hypoxic tumor microenvironment. J Exp Clin Cancer Res. 2017;36:60. 28449718 10.1186/s13046-017-0533-1 PMC5408450

[15] Wang Y , Sun H , Zhang D , et al. TP53INP1 inhibits hypoxia‐induced vasculogenic mimicry formation via the ROS/snail signalling axis in breast cancer. J Cell Mol Med. 2018;22:3475‐3488. 29655255 10.1111/jcmm.13625 PMC6010892

[16] Du J , Sun B , Zhao X , et al. Hypoxia promotes vasculogenic mimicry formation by inducing epithelial‐mesenchymal transition in ovarian carcinoma. Gynecol Oncol. 2014;133:575‐583. 24589413 10.1016/j.ygyno.2014.02.034

[17] Li W , Zong SQ , Shi Q , Li HJ , Xu J , Hou FG . Hypoxia‐induced vasculogenic mimicry formation in human colorectal cancer cells: involvement of HIF‐1a, Claudin‐4, and E‐cadherin and Vimentin. Sci Rep. 2016;6:37534. 27869227 10.1038/srep37534 PMC5116622

[18] Ahluwalia A , Tarnawski AS . Critical role of hypoxia sensor‐HIF‐1alpha in VEGF gene activation. Implications for angiogenesis and tissue injury healing. Curr Med Chem. 2012;19:90‐97. 22300081 10.2174/092986712803413944

[19] Duan L , Ye L , Zhuang L , et al. VEGFC/VEGFR3 axis mediates TGFβ1‐induced epithelial‐to‐mesenchymal transition in non‐small cell lung cancer cells. PLoS ONE. 2018;13:e0200452. 29995950 10.1371/journal.pone.0200452 PMC6040758

[20] Liu K , Sun B , Zhao X , et al. Hypoxia promotes vasculogenic mimicry formation by the Twist1‐Bmi1 connection in hepatocellular carcinoma. Int J Mol Med. 2015;36:783‐791. 26202447 10.3892/ijmm.2015.2293

[21] Wang SS , Gao XL , Liu X , et al. CD133+ cancer stem‐like cells promote migration and invasion of salivary adenoid cystic carcinoma by inducing vasculogenic mimicry formation. Oncotarget. 2016;7:29051‐29062. 27074560 10.18632/oncotarget.8665 PMC5045377

[22] Li C , Zhu M , Lou X , et al. Transcriptional factor OCT4 promotes esophageal cancer metastasis by inducing epithelial‐mesenchymal transition through VEGF‐C/VEGFR‐3 signaling pathway. Oncotarget. 2017;8:71933‐71945. 29069758 10.18632/oncotarget.18035 PMC5641101

[23] Li W , Zhou Y . LRIG1 acts as a critical regulator of melanoma cell invasion, migration and vasculogenic mimicry upon hypoxia by regulating EGFR/ERK‐triggered EMT. Biosci Rep. 2019;39:BSR20181165. 30487162 10.1042/BSR20181165 PMC6328857

[24] Liu R , Wang HL , Deng MJ , et al. Melatonin inhibits reactive oxygen species‐driven proliferation, epithelial‐mesenchymal transition, and vasculogenic mimicry in oral cancer. Oxid Med Cell Longev. 2018;2018:3510970. 29725496 10.1155/2018/3510970 PMC5884151

[25] Duan S . Silencing the autophagy‐specific gene Beclin‐1 contributes to attenuated hypoxia‐induced vasculogenic mimicry formation in glioma. Cancer Biomark. 2018;21:565‐574. 29278874 10.3233/CBM-170444 PMC13078303

[26] Li S , Zhang Q , Zhou L , et al. Inhibitory effects of compound DMBT on hypoxia‐induced vasculogenic mimicry in human breast cancer. Biomed Pharmacother. 2017;96:982‐992. 29208325 10.1016/j.biopha.2017.11.137

[27] Kim MC , Hwang SH , Kim NY , et al. Hypoxia promotes acquisition of aggressive phenotypes in human malignant mesothelioma. BMC Cancer. 2018;18:819. 30111297 10.1186/s12885-018-4720-z PMC6094475

[28] Lin SC , Chung CH , Chung CH , et al. OCT4B mediates hypoxia‐induced cancer dissemination. Oncogene. 2018;38:1093‐1105. 30209362 10.1038/s41388-018-0487-6

[29] Xu S , Zhang J , Xue H , et al. MicroRNA‐584‐3p reduces the vasculogenic mimicry of human glioma cells by regulating hypoxia‐induced ROCK1 dependent stress fiber formation. Neoplasma. 2017;64:13‐21. 27881000 10.4149/neo_2017_102

[30] Tang NN , Zhu H , Zhang HJ , et al. HIF‐1α induces VE‐cadherin expression and modulates vasculogenic mimicry in esophageal carcinoma cells. World J Gastroenterol. 2014;20:17894‐17904. 25548487 10.3748/wjg.v20.i47.17894 PMC4273139

[31] Vartanian A , Stepanova E , Grigorieva I , Solomko E , Baryshnikov A , Lichinitser M . VEGFR1 and PKCα signaling control melanoma vasculogenic mimicry in a VEGFR2 kinase‐independent manner. Melanoma Res. 2011;21:91‐98. 21389833 10.1097/CMR.0b013e328343a237

[32] Azad T , Janse van Rensburg HJ , Lightbody ED , et al. A LATS biosensor screen identifies VEGFR as a regulator of the Hippo pathway in angiogenesis. Nat Commun. 2018;9:1061. 29535383 10.1038/s41467-018-03278-w PMC5849716

[33] Xu Y , Li Q , Li XY , Yang QY , Xu WW , Liu GL . Short‐term anti‐vascular endothelial growth factor treatment elicits vasculogenic mimicry formation of tumors to accelerate metastasis. J Exp Clin Cancer Res. 2012;31:16. 22357313 10.1186/1756-9966-31-16 PMC3310846

[34] Schnegg CI , Yang MH , Ghosh SK , Hsu MY . Induction of vasculogenic mimicry overrides VEGF‐A silencing and enriches stem‐like cancer cells in melanoma. Cancer Res. 2015;75:1682‐1690. 25769726 10.1158/0008-5472.CAN-14-1855 PMC4401656

[35] Adhim Z , Matsuoka T , Bito T , et al. In vitro and in vivo inhibitory effect of three Cox‐2 inhibitors and epithelial‐to‐mesenchymal transition in human bladder cancer cell lines. Br J Cancer. 2011;105(3):393‐402. 21750550 10.1038/bjc.2011.262 PMC3172915

[36] Meng J , Chen S , Lei YY , et al. Hsp90β promotes aggressive vasculogenic mimicry via epithelial‐mesenchymal transition in hepatocellular carcinoma. Oncogene. 2018;38:228‐243. 30087438 10.1038/s41388-018-0428-4

[37] Wang H , Huang B , Li BM , et al. ZEB1‐mediated vasculogenic mimicry formation associates with epithelial‐mesenchymal transition and cancer stem cell phenotypes in prostate cancer. J Cell Mol Med. 2018;22:3768-3781. 10.1111/jcmm.13637 PMC6050489 29754422

[38] Chiang KC , Yeh CN , Pang JS , et al. 1α,25(OH)(2)D(3) analog, MART‐10, inhibits neuroendocrine tumor cell metastasis after VEGF‐A stimulation. Anticancer Res. 2017;37:6215‐6221. 29061804 10.21873/anticanres.12072

[39] Kim M , Jang K , Miller P , et al. VEGFA links self‐renewal and metastasis by inducing Sox2 to repress miR‐452, driving Slug. Oncogene. 2017;36:5199‐5211. 28504716 10.1038/onc.2017.4 PMC5596211

[40] Besharat ZM , Sabato C , Po A , et al. Low expression of miR‐466f‐3p sustains epithelial to mesenchymal transition in sonic hedgehog medulloblastoma stem cells through Vegfa‐Nrp2 signaling pathway. Front Pharmacol. 2018;9:1281. 30483126 10.3389/fphar.2018.01281 PMC6240675

[41] Mani SA , Guo W , Liao MJ , et al. The epithelial‐mesenchymal transition generates cells with properties of stem cells. Cell. 2008;133:704‐715. 18485877 10.1016/j.cell.2008.03.027 PMC2728032

[42] Chen C , Okita Y , Watanabe Y , et al. Glycoprotein nmb is exposed on the surface of dormant breast cancer cells and induces stem cell‐like properties. Cancer Res. 2018;78:6424‐6435. 30224376 10.1158/0008-5472.CAN-18-0599

[43] Fan YL , Zheng M , Tang YL , Liang XH . A new perspective of vasculogenic mimicry: EMT and cancer stem cells (Review). Oncol Lett. 2013;6:1174‐1180. 24179490 10.3892/ol.2013.1555 PMC3813799

[44] Jang K , Kim M , Gilbert CA , Simpkins F , Ince TA , Slingerland JM . VEGFA activates an epigenetic pathway upregulating ovarian cancer‐initiating cells. EMBO Mol Med. 2017;9:304‐318. 28179359 10.15252/emmm.201606840 PMC5331266

[45] Ramezani S , Vousooghi N , Kapourchali FR , et al. Rolipram potentiates bevacizumab‐induced cell death in human glioblastoma stem‐like cells. Life Sci. 2017;173:11‐19. 28202289 10.1016/j.lfs.2017.02.005

[46] Wang H , Lin H , Pan J , et al. Vasculogenic mimicry in prostate cancer: the roles of EphA2 and PI3K. J Cancer. 2016;7:1114‐1124. 27326255 10.7150/jca.14120 PMC4911879

[47] Williamson SC , Metcalf RL , Trapani F , et al. Vasculogenic mimicry in small cell lung cancer. Nat Commun. 2016;7:13322. 27827359 10.1038/ncomms13322 PMC5105195

[48] You X , Wang Y , Wu J , et al. Prognostic significance of galectin‐1 and vasculogenic mimicry in patients with gastric cancer. Onco Targets Ther. 2018;11:3237‐3244. 29881296 10.2147/OTT.S165899 PMC5985771

