# Supplementary figures and images for: Hypoxia promotes vasculogenic mimicry formation by vascular endothelial growth factor A mediating epithelial‐mesenchymal transition in salivary adenoid cystic carcinoma
Source: Cell Prolif. 2019 Apr 3;52(3):e12600. doi: 10.1111/cpr.12600 (PMC6536414; doi:10.1111/cpr.12600)

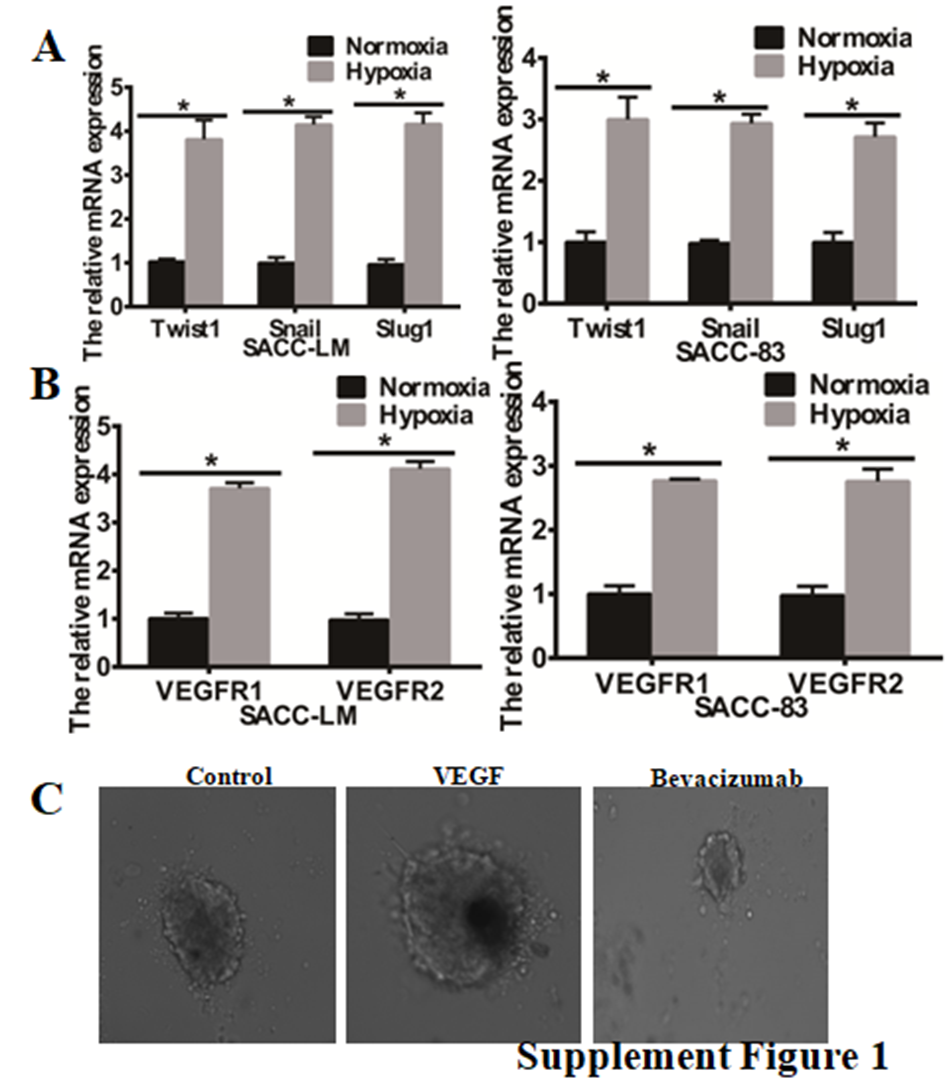

Supplement: Supplementary file 1 [file CPR-52-e12600-s001.tif]
